# Prediction of gait subtypes in children with bilateral spastic cerebral palsy using Edinburgh Visual Gait Score

**DOI:** 10.3389/fpubh.2026.1884116

**Published:** 2026-08-10

**Authors:** Nan Tong, Turong Chen, Long Xu, Li Gao, Xue Bai, Guixiang Liu, Dongxue Zhang, Guojun Yun, Meihuan Huang

**Affiliations:** 1 Department of Rehabilitation Medicine, Shenzhen Children’s Hospital, Shenzhen, China; 2 Department of Pediatric Rehabilitation, Xiangya Boai Rehabilitation Hospital, Changsha, China; 3 Department of Rehabilitation Sciences, Faculty of Health and Social Sciences, The Hong Kong Polytechnic University, Kowloon, Hong Kong SAR, China

**Keywords:** Edinburgh Visual Gait Score, gait classification, machine learning, observational gait analysis, pediatric rehabilitation, Random Forest, spastic bilateral cerebral palsy

## Abstract

**Objectives:**

Early and accurate gait subtype classification in children with spastic bilateral cerebral palsy (CP) is essential for guiding rehabilitation. While three-dimensional gait analysis is limited by cost and accessibility, the Edinburgh Visual Gait Score (EVGS) provides a feasible observational tool. This study evaluates the discriminative value of EVGS-derived features using machine learning to support gait phenotype stratification.

**Methods:**

This retrospective study included 40 children with spastic bilateral CP (80 lower limbs). EVGS item-level and subscale scores were extracted from standardized gait videos. Gait patterns were classified into four pathological subtypes by two blinded raters. A Random Forest (RF) classifier was evaluated using 5-fold stratified group cross-validation, with Multinomial Logistic Regression (MLR) serving as a benchmark. To address within-subject dependency, a sensitivity analysis involving random single-limb sampling was performed. Model performance was assessed using balanced accuracy, macro-averaged F1-score, area under the curve (AUC), and class-specific sensitivity, specificity, precision, recall, and F1-score. Feature importance was analyzed by permutation and Gini metrics.

**Results:**

The RF model using item-level features outperformed MLR, achieving a mean balanced accuracy of 0.64 (SD = 0.03) and a macro-AUC of 0.74 (SD = 0.05). Item-level data consistently outperformed aggregated subscale scores. Sensitivity analysis using random single-limb sampling confirmed the robustness of these findings, yielding comparable performance estimates. For class-specific performance, the model achieved moderate discriminative ability for Type 1 (AUC = 0.76) and Type 3 (AUC = 0.69), limited performance for Type 2 (AUC = 0.55) due to feature overlap, and high discriminative ability for Type 4 (AUC = 0.89). Feature importance analysis identified hip peak flexion at swing (Item 13), knee position at terminal swing (Item 10), knee peak extension at stance (Item 9), trunk peak sagittal position (Item 16), and maximum ankle dorsiflexion at swing (Item 7) as key predictors.

**Conclusion:**

EVGS item-level features facilitate moderate yet robust gait subtype classification in spastic bilateral CP. The integration of EVGS with machine learning presents a promising adjunctive approach for preliminary assessment, particularly in resource-limited settings; however, external validation is requisite prior to clinical implementation.

## 1 Introduction

Bilateral spastic cerebral palsy (CP), commonly manifesting as spastic diplegia or quadriplegia, is one of the most prevalent CP subtypes, characterized by bilateral lower-limb involvement with spasticity, muscle weakness, impaired selective motor control, and poor postural stability, resulting in abnormal gait patterns and reduced functional mobility (1). Gait abnormalities in spastic bilateral CP are highly heterogeneous, with common patterns including crouch gait, jump gait, true equinus, and apparent equinus, each reflecting different combinations of neuromuscular impairments and compensatory mechanisms (2). Accurate classification of these gait subtypes is essential for guiding individualized treatment strategies, including surgical decision-making, orthotic prescription, and targeted rehabilitation interventions (3, 4).

The gait classification system for sagittal patterns in bilateral spastic CP proposed by Rodda and Graham is among the most widely adopted frameworks (5). It categorizes gait patterns based on sagittal plane kinematics of the ankle, knee, hip, and pelvis into four main types: true equinus, jump gait, apparent equinus, and crouch gait (5). This system has demonstrated strong clinical utility and good inter-rater reliability when applied to instrumented three-dimensional gait analysis (3DGA) data (5–7). Despite some variability in the definitions of the four multi-joint patterns, the Rodda classification is regarded as one of the most comprehensive, as it explicitly incorporates co-occurring deviations across all lower-limb joints and builds upon the gait classification system (GCS) with established validity and reliability (2). However, 3DGA remains resource-intensive, requiring specialized laboratories, trained personnel, and significant financial investment, thereby limiting its accessibility in low-resource settings and routine clinical practice (8). Consequently, there is a pressing need for scalable, cost-effective classification tools that maintain diagnostic accuracy without reliance on complex biomechanical instrumentation.

The Edinburgh Visual Gait Score (EVGS) was developed as a standardized observational gait assessment tool to address this gap (9). Comprising 17 items across six anatomical domains (foot, knee, hip, pelvis, trunk, and total score), EVGS demonstrates robust validity, good intra-and inter-observer reliability, and strong correlation with 3DGA parameters (10, 11). Its simplicity, minimal equipment requirements, and compatibility with routine video recording have facilitated widespread adoption in clinical and community-based settings (12). Despite acceptable validity and reliability, the EVGS has mainly been used for descriptive assessment and longitudinal monitoring rather than gait subtype classification or prediction. Its interpretation still relies on clinician judgment and rule-based scoring, which may reduce objectivity and limit discrimination between overlapping or transitional gait patterns, particularly in mild cases. These limitations underscore the need for data-driven approaches leveraging EVGS-derived features for automated gait subtype prediction in bilateral spastic CP.

Recent advances in machine learning (ML) have substantially improved gait pattern recognition in cerebral palsy. Supervised models, including Random Forests, support vector machines, artificial neural networks (ANNs), k-nearest neighbors (KNN), convolutional neural networks (CNNs), and long short-term memory (LSTM) networks, have been applied to classify gait subtypes using 3DGA) kinematic and kinetic data, with reported accuracies ranging from 0.78 to 0.93 (13–15). These approaches exploit high-dimensional feature spaces to capture non-linear inter-joint coordination patterns that are difficult to identify using conventional visual assessment (13). However, most existing ML-based gait studies remain restricted to laboratory-based 3DGA datasets, limiting their clinical translation (15). Importantly, no study has systematically investigated whether EVGS-derived scores, which reflect clinically relevant gait deviations across key anatomical domains, can be used as input features for ML-based gait subtype prediction. Given their direct correspondence with established gait classification frameworks, integrating EVGS with ML methods may provide a feasible and clinically scalable approach to automated gait phenotyping in bilateral spastic cerebral palsy.

This study aims to investigate the ability of EVGS item-level and subtotal scores to discriminate gait subtypes in children with spastic bilateral CP. We hypothesize that EVGS-derived features contain sufficient discriminative information to differentiate clinically defined gait patterns. Machine learning classifiers will be used as analytical tools to evaluate classification performance and identify the most informative EVGS items. This approach seeks to assess the clinical utility of EVGS for gait subtype differentiation and its potential to support decision-making in routine practice, particularly in resource-limited settings.

## 2 Methods

### 2.1 Study design and participants

This retrospective methodological study included children with bilateral spastic CP who underwent clinical gait assessment at the Rehabilitation Center of Shenzhen Children’s Hospital. A total of 40 participants (80 lower limbs) were included in the final analysis. Each limb was treated as an independent analytical unit due to the commonly observed asymmetry in gait patterns among children with bilateral spastic CP. Participant demographic and clinical characteristics, including age, sex, motor subtype, and Gross Motor Function Classification System (GMFCS) level, were collected and summarized. This study was approved by the Institutional Review Board of Shenzhen Children’s Hospital (No. 202106202). Written informed consent was obtained from the legal guardians of all participants prior to data collection.

Participants were included if they met the following criteria: (1) diagnosis of bilateral spastic cerebral palsy confirmed by a pediatric rehabilitation specialist; (2) age between 3 and 17 years; and (3) availability of at least one complete EVGS assessment. Participants were excluded if they had: (1) a history of orthopedic surgery or botulinum toxin injection within 6 months prior to assessment; (2) severe cognitive impairment preventing cooperation during gait evaluation; or (3) incomplete gait or EVGS records.

### 2.2 Gait assessment and EVGS scoring

Gait patterns were assessed using the EVGS, a validated 17-item observational gait analysis tool widely used in children with cerebral palsy (9). EVGS evaluates kinematic deviations across multiple anatomical regions, including the foot and ankle, knee, hip, pelvis, and trunk, based on predefined observational criteria in the sagittal and frontal planes.

Standardized clinical video recordings were acquired in a dedicated gait assessment setting. Participants were instructed to walk barefoot at a self-selected comfortable speed along a 10-meter walkway. At least two gait cycles were recorded using high-definition cameras positioned in standardized sagittal and coronal planes. The most representative trials were selected for analysis. All recordings were anonymized and processed offline. Selected videos were imported into Kinovea software (version 2023.1) for slow-motion review and joint angle estimation, supporting detailed qualitative and quantitative evaluation of gait kinematics for EVGS scoring.

Each limb was independently scored using standardized EVGS criteria by one of two experienced physical therapists (>10 years of gait analysis experience). The total EVGS score was calculated as the sum of all items, with higher scores indicating greater gait deviation. Subscale scores were derived for the foot and ankle, knee, hip, pelvis, and trunk.

Gait subtype labeling was conducted in a separate session from EVGS scoring to minimize incorporation bias. According to the sagittal gait classification framework described by Rodda et al. (5), each limb was classified into one of four pathological gait subtypes, namely true equinus, jump gait, apparent equinus, and crouch gait, with an additional mild/within normal limits (WNL) category included for limbs without a clear pathological subtype. Two experienced physical therapists independently classified all limbs. Raters were blinded to the EVGS total or subscale scores when assigning gait subtype labels. The initial percentage agreement between the two raters was 82.5%, and Cohen’s kappa was 0.76, indicating substantial inter-rater agreement. Fourteen limbs required adjudication, and disagreements were resolved by a senior evaluator to determine the final consensus reference labels used for model development and evaluation. Only limbs with pathological gait patterns were included in the machine learning classification models.

### 2.3 Statistical analysis

All statistical analyses were performed using R software version 4.5.1. EVGS-derived variables served as candidate predictors for gait subtype classification. To examine the influence of feature granularity and redundancy, three feature sets were evaluated: (1) an item-only model (17 individual EVGS items); (2) a subscale-only model (domain scores for foot/ankle, knee, hip, pelvis, and trunk); and (3) a combined model (both item-level and subscale scores). All continuous predictors were standardized prior to model training.

The primary classifier was a Random Forest (RF) model implemented using the randomForest package. RF was selected for its ability to capture nonlinear relationships and interactions among gait features while providing interpretability through feature importance metrics. Given the limited sample size, extensive hyperparameter tuning was avoided to prevent overfitting. The number of trees was set to 500 to stabilize ensemble estimates, and mtry was kept at the default value for classification. Other parameters remained at their default settings. For benchmarking, a Multinomial Logistic Regression (MLR) model was also trained using the same feature sets and validation strategy.

Model performance was evaluated using stratified 5-fold cross-validation at the participant level. This approach ensured that both limbs from the same participant were assigned to the same fold, thereby preventing data leakage between training and testing sets. In each iteration, four folds were used for training and one for testing. Final performance estimates were averaged across the five folds. No oversampling or undersampling was applied to address class imbalance, as this could introduce instability in small datasets; instead, balanced accuracy and class-specific metrics were prioritized.

Classification performance was assessed using overall accuracy, balanced accuracy, macro-averaged F1-score, and class-specific sensitivity, specificity, precision, recall, and F1-score. Multiclass receiver operating characteristic (ROC) analysis was performed using a one-vs-rest approach (pROC package), with Area Under the Curve (AUC) calculated for each subtype and macro-averaged across classes. Where appropriate, 95% confidence intervals (CIs) were estimated to reflect uncertainty due to the small sample size.

Feature importance was primarily assessed using Mean Decrease in Accuracy (permutation importance), which measures the reduction in model performance when a feature’s values are randomly shuffled. Mean Decrease in Gini Impurity was calculated as a complementary metric to validate the consistency of feature rankings.

To address potential within-subject dependency between bilateral limbs, a sensitivity analysis was conducted. In this procedure, one limb per participant was randomly selected across 1,000 iterations, and the model performance was re-evaluated to ensure that the primary findings were not driven by data redundancy.

## 3 Results

### 3.1 Characteristics of the study population

A total of 40 children with bilateral spastic BCP were included, contributing 80 lower limbs for analysis. The mean age was 8.7 ± 3.0 years (range: 4–15 years). Most participants had spastic diplegia (90%) and were classified as GMFCS levels I–II. Detailed demographic characteristics and EVGS total scores are presented in Table 1.

**Table 1:** Characteristics of participants.

Variables	*n* (%) or Mean (SD)	EVGS total score, Mean (SD)
*N*	40	
Number of limbs (*n*)	80	
Age, years		
Mean (SD)	8.7 (3.0)	
Range	4–15	
Sex		
Male	24 (60.0%)	12.3 (5.74)
Female	16 (40.0%)	11.3 (4.93)
Motor types		
Diplegia	36 (90.0%)	11.4 (5.42)
Quadriplegia	4 (10.0%)	15.9 (3.76)
GMFCS levels		
Level I	16 (40.0%)	7.7 (3.12)
Level II	24 (60.0%)	14.7 (4.8)
Asymmetry	18 (45.0%)	
Orthoses		
AFO	9 (22.5%)	
UCBL	6 (12.5%)	

SD, standard deviation; GMFCS, Gross Motor Function Classification System; EVGS, Edinburgh Visual Gait Score; AFO, Ankle-Foot Orthosis; UCBL, University of California Biomechanics Laboratory (shoe insert/orthosis).

### 3.2 EVGS-derived gait pattern characteristics

**Figure 1:**
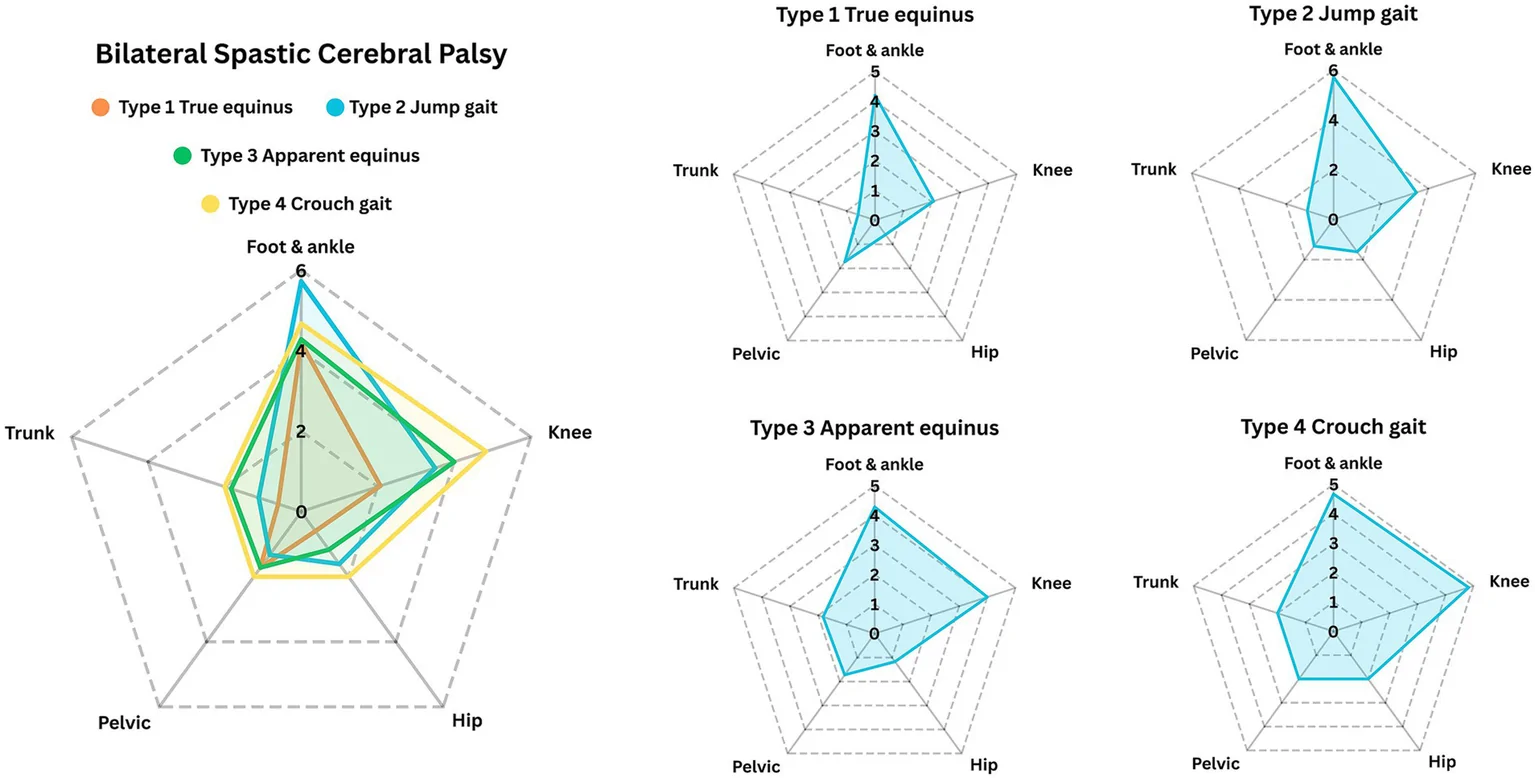
EVGS profiles for gait pattern subtypes in bilateral spastic cerebral palsy. EVGS, Edinburgh Visual Gait Score.

The distribution of EVGS total and subdomain scores across gait subtypes is shown in Table 2, and EVGS profiles are illustrated in Figure 1. EVGS total scores demonstrated an increasing trend from WNL to crouch gait. Significant differences among gait subtypes were observed for EVGS total score and most subdomains, including foot and ankle, knee, hip, and trunk (all *p* < 0.001), whereas pelvic scores were not statistically different (*p* = 0.124).

**Table 2:** Gait subtypes and EVGS profiles in bilateral spastic cerebral palsy (*n* = 80).

Gait patterns	*n*	Total	Foot & ankle	Knee	Hip	Pelvic	Trunk
WNL^*^	9	4.89 (2.03)	1.67 (1.32)	1.78 (0.441)	0.667 (1.32)	0.78 (0.83)	0.00 (0.00)
Type 1	18	9.67 (4.07)	4.44 (1.65)	2.22 (1.35)	0.56 (0.78)	1.61 (0.78)	0.83 (1.29)
Type 2	25	13.2 (5.07)	5.16 (2.32)	3.64 (1.60)	1.76 (1.27)	1.40 (1.08)	1.24 (1.27)
Type 3	20	13.2 (4.99)	4.20 (1.79)	4.20 (1.67)	1.35 (1.18)	1.75 (1.02)	1.65 (1.23)
Type 4	8	17.5 (3.02)	5.50 (1.69)	5.38 (1.06)	2.75 (1.04)	1.63 (0.744)	2.25 (0.707)
Kruskal-Wallis test	ϵ^2^	0.39	0.23	0.37	0.24	0.04	0.25
	*p*	<0.001	<0.001	<0.001	<0.001	0.124	<0.001

WNL, mild or within normal limits; EVGS, Edinburgh Visual Gait Score. Data are presented as mean ± standard deviation. Type 1, true equinus; Type 2, jump gait; Type 3, apparent equinus; Type 4, crouch gait. * The WNL group was included for descriptive comparison, but excluded from the machine learning classification models.

### 3.3 Classification performance and feature set comparison

We evaluated the classification performance of RF and MLR models using three distinct feature sets: item-level items only, subscale scores only, and a combined set (Table 3). The RF model using item-level features achieved the highest mean balanced accuracy (0.64, SD = 0.03), macro F1 (0.52, SD = 0.09), and macro-AUC (0.74, SD = 0.05). Detailed performance metrics for each cross-validation fold are provided in Supplementary Table S1. In contrast, aggregating items into subscales did not improve RF performance; the combined and subscale-only sets yielded lower balanced accuracies (0.58 and 0.53, respectively). MLR showed a different trend, with higher balanced accuracy for subscale scores (0.61) than for individual items (0.55). Overall, MLR models underperformed compared to RF, with the best MLR configuration (subscale-only) achieving a macro-AUC of 0.68.

**Table 3:** Cross-validation performance metrics for Random Forest and Multinomial Logistic Regression models across different feature sets.

Model	Feature set	Accuracy	Balanced accuracy	Macro F1	Macro AUC
RF	Item-only	0.47 (0.07)	**0.64 (0.03)**	**0.52 (0.09)**	**0.74 (0.05)**
	Subscale-only	0.32 (0.11)	0.53 (0.07)	0.37 (0.10)	0.71 (0.13)
	Combined	0.39 (0.10)	0.58 (0.06)	0.42 (0.08)	0.74 (0.08)
MLR	Item-only	0.33 (0.12)	0.55 (0.07)	0.36 (0.08)	0.64 (0.11)
	Subscale-only	0.44 (0.12)	0.61 (0.07)	0.49 (0.14)	0.68 (0.10)
	Combined	0.40 (0.13)	0.61 (0.09)	0.43 (0.13)	0.65 (0.09)

RF, Random Forest; MLR, Multinomial Logistic Regression; AUC, area under the curve. Data were presented as mean (SD). Bold values indicate the best-performing model metrics.

### 3.4 Class-specific classification metrics

**Figure 2:**
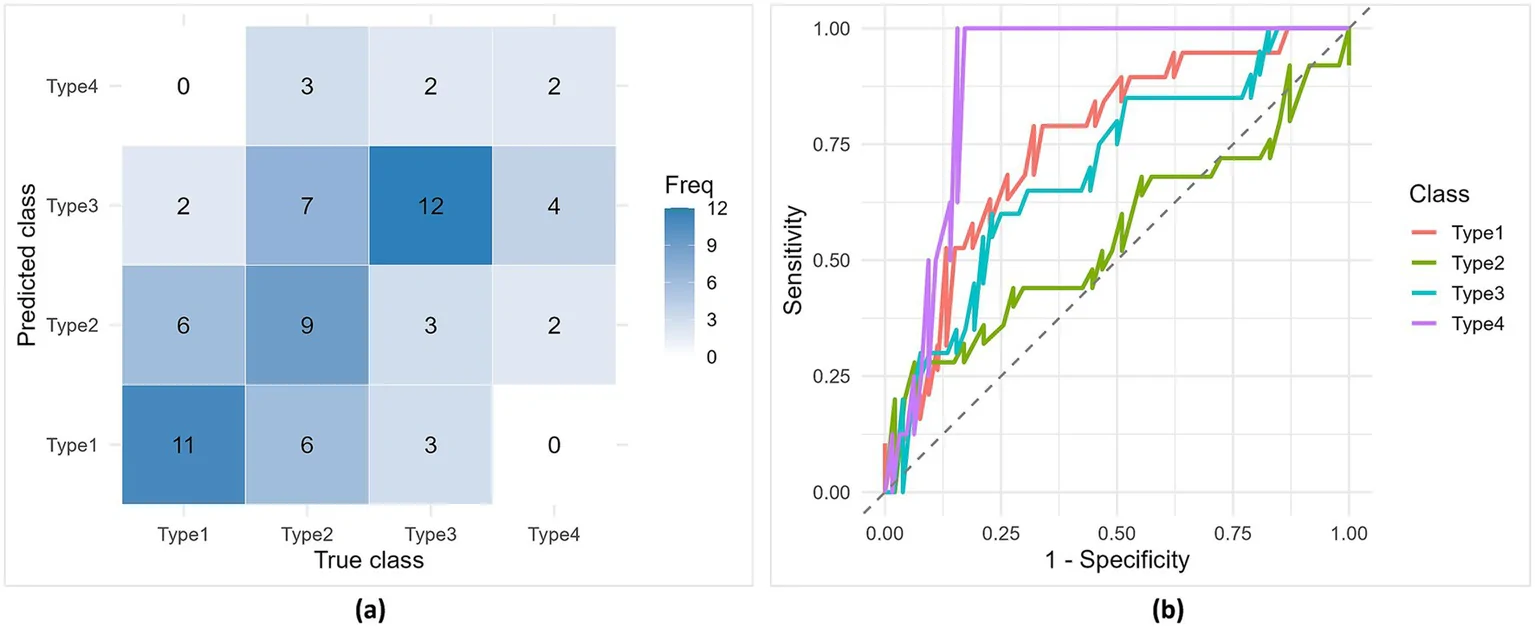
Confusion matrix and ROC curves of EVGS item-based gait subtype classification. **(a)** Confusion matrix. **(b)** ROC curves. EVGS, Edinburgh Visual Gait Score; ROC, receiver operating characteristic; AUC, area under the curve.

Table 4 details the class-specific performance of the optimal RF model (item-level features), with corresponding ROC curves presented in Figure 2. The model demonstrated moderate discriminative ability for Type 1 (sensitivity = 0.58; balanced accuracy = 0.71; AUC = 0.76) and Type 3 (sensitivity = 0.60; AUC = 0.69), although Type 3 exhibited lower precision (0.40). As shown in the confusion matrix (Figure 2a), Type 2 was the most challenging to classify. It yielded the lowest sensitivity (0.36), balanced accuracy (0.56), and AUC (0.55), suggesting substantial feature overlap with other gait patterns. For the minority class Type 4, the model achieved modest sensitivity (0.25) but high specificity (0.92) and the highest AUC (0.89). This indicates that while few Type 4 cases were correctly identified, false positives were rare, though these results should be interpreted with caution due to the limited sample size (*n* = 8).

**Table 4:** Classification performance by gait type for the Random Forest model with item-level features.

Gait class	*n*	Sensitivity	Specificity	Precision	F1 Score	Balanced accuracy	AUC (95%CI)
Type 1	19	0.58	0.83	0.55	0.56	0.71	0.76 (0.63,0.88)
Type 2	25	0.36	0.77	0.45	0.40	0.56	0.55 (0.40, 0.70)
Type 3	20	0.60	0.75	0.48	0.53	0.68	0.69 (0.56, 0.83)
Type 4	8	0.25	0.92	0.29	0.27	0.59	0.89 (0.82, 0.97)

### 3.5 Feature importance analysis

**Figure 3:**
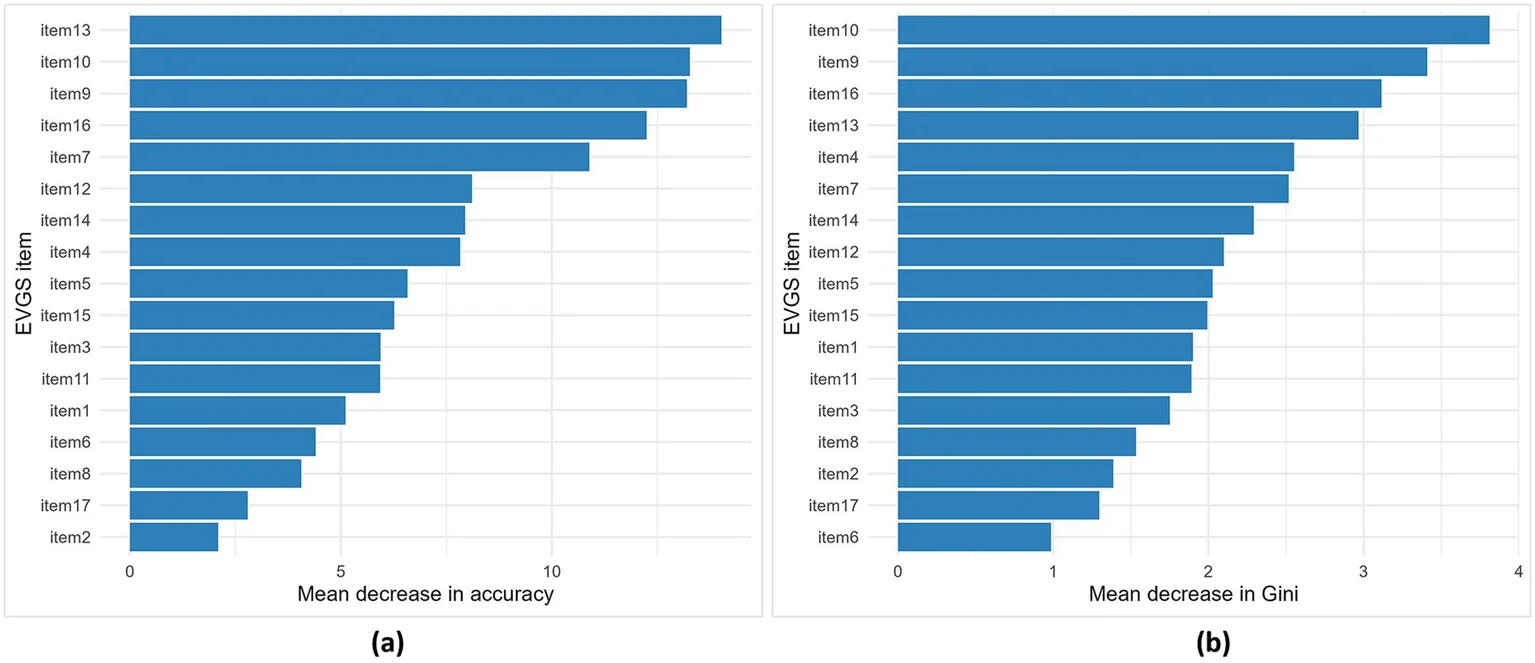
Feature importance of EVGS item scores in gait subtype classification. **(a)** Permutation importance; **(b)** Gini importance. EVGS, Edinburgh Visual Gait Score.

Primary feature importance analysis based on permutation importance (Mean Decrease in Accuracy) identified hip peak flexion at swing (Item 13; 14.02), knee position at terminal swing (Item 10; 13.27), knee peak extension at stance (Item 9; 13.20), trunk peak sagittal position (Item 16; 12.24), and maximum ankle dorsiflexion at swing (Item 7; 10.89) as the most influential predictors (Figure 3a). This ranking was largely corroborated by Mean Decrease in Gini impurity analysis, which similarly highlighted Items 10, 9, 16, and 13 among the top contributors (Figure 3b), reinforcing the robustness of these key biomechanical features.

### 3.6 Sensitivity analysis: single-limb sampling

To address potential within-subject dependency, we performed a sensitivity analysis in which only one limb per child was randomly selected across 1,000 iterations. This procedure yielded a mean balanced accuracy of 0.60 (SD = 0.05) and a macro F1 score of 0.46 (SD = 0.08). The main conclusions remained unchanged, although performance estimates were relatively lower and exhibited wider variability compared to the primary analysis. These findings support the exploratory nature of the study while confirming that the observed classification patterns are robust to the removal of bilateral data redundancy.

## 4 Discussion

This study investigated the discriminative value of EVGS-derived features for gait subtype classification in children with spastic bilateral CP using a machine learning approach. The results demonstrated that EVGS item-level scores contain sufficient information to differentiate clinically defined gait patterns, achieving moderate classification performance with robust discriminative ability. These findings support the clinical relevance of structured observational gait assessment for phenotype stratification beyond purely descriptive use.

A key finding was the discrepancy between high discriminative ability and moderate balanced accuracy. This suggests that while the model can effectively rank the probability of gait subtypes, assigning definitive categorical labels remains challenging. Misclassifications occurred primarily between adjacent patterns (e.g., true equinus, jump gait, and apparent equinus), consistent with the recognized continuum of sagittal plane deformities in bilateral spastic CP (2). These overlapping phenotypes reflect shared pathomechanical mechanisms, including progressive muscle–tendon imbalance and altered joint kinetics, which limit clear categorical separation even in instrumented gait analysis (13). Furthermore, the superior performance of granular item-level data over aggregated subscale scores indicates that preserving detailed observational signals is critical, as aggregation may dilute subtle kinematic differences essential for distinguishing adjacent phenotypes.

Feature importance analysis further elucidated the biomechanical basis of this discrimination. Permutation importance identified knee terminal swing, knee stance extension, hip swing flexion, trunk sagittal position, and ankle dorsiflexion as the most influential predictors, a ranking corroborated by Gini impurity metrics. This convergence highlights that multi-joint coordination involving the ankle, knee, hip, and trunk is central to the model’s discriminative ability. However, these metrics should be interpreted cautiously; they reflect predictive influence rather than causal biomechanical relationships and may be subject to biases related to feature correlation or scale.

Despite these meaningful findings, the observed performance ceiling likely stems from inherent limitations of the EVGS framework. As an observational system, EVGS evaluates joint deviations independently without explicitly capturing inter-joint coordination patterns (9). Since gait subtype differentiation is largely driven by coupled multi-joint kinematics, the lack of explicit interaction features may constrain the model’s capacity to fully represent underlying biomechanics (16). In addition, gait abnormalities in spastic bilateral CP often exist along a continuous spectrum rather than discrete categories, with substantial overlap in joint-level impairments across adjacent gait patterns (2, 16, 17).

Compared with 3DGA-based studies reporting accuracies of 0.78–0.93 (13–15), our lower performance reflects the trade-off between clinical feasibility and measurement precision. While 3DGA benefits from high-dimensional kinematic and kinetic data that capture complex inter-joint coordination, EVGS is a low-cost, clinically feasible tool that summarizes gait deviations across key anatomical regions (18). Although EVGS does not explicitly encode inter-joint coupling, our results suggest it retains sufficient biomechanical information to support gait subtype discrimination when combined with machine learning. Given that the high cost and technical demands of 3DGA restrict its routine use, particularly in resource-limited settings (19), EVGS offers a viable proxy for gait phenotype identification when enhanced by machine learning.

From a translational perspective, the integration of EVGS with machine learning–based classification may provide a pragmatic pathway for enhancing the utility of observational gait assessment. Rather than replacing instrumented gait analysis or clinical expertise, this approach may serve as a complementary decision-support tool to improve consistency in gait interpretation and assist clinicians in identifying patients who may benefit from targeted interventions, including orthotic prescription, physical therapy, or surgical planning (20). However, given its current exploratory nature, the proposed model should be viewed as an adjunct to, rather than a substitute for, comprehensive clinical assessment. Larger prospective multicenter studies with external validation are essential to establish robustness before clinical implementation can be considered.

Several limitations warrant careful consideration when interpreting these preliminary exploratory findings. First, the single-center design, relatively small sample size, and uneven class distribution particularly for minority subtypes such as Type 4 limit the generalizability of the results and likely contributed to the lower sensitivity observed for rare classes. Second, although raters were blinded to EVGS scores during labeling, the retrospective nature of the study means that subtle biases in video selection or interpretation cannot be entirely excluded. Third, despite the use of stratified group cross-validation and sensitivity analyses to address within-subject dependency, the potential correlation between bilateral limbs remains a statistical challenge. However, our sensitivity analysis confirmed that the main conclusions are robust to this limitation. Fourth, the absence of an external validation cohort means that the model’s performance on unseen populations from different centers remains unknown. Finally, while EVGS provides a standardized observational framework, it lacks the kinetic and high-resolution kinematic data of 3DGA, which remains the gold standard for capturing complex inter-joint dynamics. Consequently, the current model should be viewed as a complementary screening tool rather than a replacement for comprehensive instrumented gait analysis.

## 5 Conclusion

This study provides preliminary evidence that EVGS-derived features contain clinically meaningful information that can support gait subtype classification in children with spastic bilateral cerebral palsy. While not yet ready for standalone clinical deployment, combining structured observational gait assessment with data-driven analytical methods offers a pragmatic pathway to enhance the utility of EVGS in pediatric neurorehabilitation, particularly for triage and decision support in settings with limited access to advanced gait laboratories.

## Data Availability

The data that support the findings of this study are available from the corresponding author upon reasonable request.

## References

[1] Novak I Hines M Goldsmith S Barclay R. Clinical prognostic messages from a systematic review on cerebral palsy. Pediatrics. (2012) 130:e1285–312. doi: 10.1542/peds.2012-0924, 23045562

[2] Papageorgiou E Nieuwenhuys A Vandekerckhove I Van Campenhout A Ortibus E Desloovere K. Systematic review on gait classifications in children with cerebral palsy: an update. Gait Posture. (2019) 69:209–23. doi: 10.1016/j.gaitpost.2019.01.038, 30851621

[3] Davids JR Bagley AM. Identification of common gait disruption patterns in children with cerebral palsy. J Am Acad Orthop Surg. (2014) 22:782–90. doi: 10.5435/jaaos-22-12-782, 25425613

[4] Armand S Decoulon G Bonnefoy-Mazure A. Gait analysis in children with cerebral palsy. EFORT Open Rev. (2016) 1:448–60. doi: 10.1302/2058-5241.1.000052 28698802 PMC5489760

[5] Rodda J Graham HK Carson L Galea MP Wolfe R. Sagittal gait patterns in spastic diplegia. J Bone Joint Surg Br. (2004) 86-B:251–8. doi: 10.1302/0301-620X.86B2.13878, 15046442

[6] Stott NS Atherton WG Mackey AH Galley IJ Nicol RO Walsh SJ. The reliability and validity of assessment of sagittal plane deviations in children who have spastic Diplegia. Arch Phys Med Rehabil. (2005) 86:2337–41. doi: 10.1016/j.apmr.2005.06.021, 16344032

[7] Morais Filho MC Kawamura CM Lopes JAF Neves DL Cardoso MO Caiafa JB. Most frequent gait patterns in diplegic spastic cerebral palsy. Acta Ortop Bras. (2014) 22:197–201. doi: 10.1590/1413-78522014220400942, 25246849 PMC4167043

[8] Salem Y Krzak JJ Godwin EM McMulkin ML Kaplan SL. Three-dimensional instrumented gait analysis for children with cerebral palsy: an evidence-based clinical practice guideline. Pediatr Phys Ther. (2024) 36:182–206. doi: 10.1097/PEP.0000000000001101, 38568266

[9] Read HS Hazlewood ME Hillman SJ Prescott RJ Robb JE. Edinburgh visual gait score for use in cerebral palsy. J Pediatr Orthop. (2003) 23:296–301. doi: 10.1097/01241398-200305000-00005, 12724590

[10] Ong A Hillman S Robb J. Reliability and validity of the Edinburgh visual gait score for cerebral palsy when used by inexperienced observers. Gait Posture. (2008) 28:323–6. doi: 10.1016/j.gaitpost.2008.01.008, 18328710

[11] Orozco M d PD Abousamra O Church C Lennon N Henley J Rogers KJ . Reliability and validity of Edinburgh visual gait score as an evaluation tool for children with cerebral palsy. Gait Posture. (2016) 49:14–8. doi: 10.1016/j.gaitpost.2016.06.017, 27344448

[12] Tzikalagia T Ramdharry G. Using the Edinburgh visual gait score to assess gait in children with cerebral palsy: a feasibility evaluation. Int J Ther Rehabil. (2017) 24:419–26. doi: 10.12968/IJTR.2017.24.10.419

[13] Zhang Y Ma Y. Application of supervised machine learning algorithms in the classification of sagittal gait patterns of cerebral palsy children with spastic diplegia. Comput Biol Med. (2019) 106:33–9. doi: 10.1016/j.compbiomed.2019.01.009, 30665140

[14] Kavya Y Reka SS, editors. Classification of gait in children suffering from cerebral palsy using machine learning and deep learning techniques 2024 IEEE 1st International Conference on Green Industrial Electronics and Sustainable Technologies (GIEST); (2024) Imphal, India: IEEE.

[15] Kavya Y Reka SS. Abnormal gait classification in children with cerebral palsy using convLSTM hybrid model and GAN. IEEE Access. (2024) 12:117721–36. doi: 10.1109/ACCESS.2024.3439889

[16] Dobson F Morris ME Baker R Graham HK. Gait classification in children with cerebral palsy: a systematic review. Gait Posture. (2007) 25:140–52. doi: 10.1016/j.gaitpost.2006.01.003, 16490354

[17] Melanda AG Davids JR Pauleto AC Pelegrinelli AR Ferreira AEK Knaut LA . Reliability and validity of the gait classification system in children with cerebral palsy (GCS-CP). Gait Posture. (2022) 98:355–61. doi: 10.1016/j.gaitpost.2022.09.083, 36308864

[18] Karibzhanova D Jamwal PK Khawaja AR Kystaubayeva Z Kapsalyamov A Shakenov M . Clinical and instrument-based assessment of balance, gait, and motor functions in pediatric cerebral palsy: a systematic review. Wearable Technol. (2025) 6:e29. doi: 10.1017/wtc.2025.10011, 40692806 PMC12277211

[19] States RA Krzak JJ Salem Y Godwin EM Bodkin AW McMulkin ML. Instrumented gait analysis for management of gait disorders in children with cerebral palsy: a scoping review. Gait Posture. (2021) 90:1–8. doi: 10.1016/j.gaitpost.2021.07.009, 34358847

[20] Kija E Namata TT Sebunya R. Management of cerebral palsy in low-and middle-income countries. Semin Pediatr Neurol. (2025) 55:101202. doi: 10.1016/j.spen.2025.101202, 41076299

