# Supplementary material for: Prediction of gait subtypes in children with bilateral spastic cerebral palsy using Edinburgh Visual Gait Score
Source: Front Public Health. 2026 Aug 10;14:1884116. doi: 10.3389/fpubh.2026.1884116 (PMC13501382; doi:10.3389/fpubh.2026.1884116)
Supplement: Supplementary file 1 [file Table_1.DOCX]

**Table S1.** Performance metrics for the Random Forest (Item-only) model across five folds of stratified group cross-validation.

| Fold | Accuracy | Balanced Accuracy | Macro F1 | Macro AUC |
| --- | --- | --- | --- | --- |
| 1 | 0.39 | 0.62 | 0.51 | 0.68 |
| 2 | 0.44 | 0.61 | 0.44 | 0.76 |
| 3 | 0.50 | 0.65 | 0.48 | 0.74 |
| 4 | 0.56 | 0.68 | 0.64 | 0.79 |
| 5 | 0.44 | 0.63 | 0.43 | 0.73 |
